# Artemisinin derivative-containing therapies and abnormal hemoglobin: Do we need to adapt the treatment?

**DOI:** 10.1051/parasite/2021063

**Published:** 2021-09-27

**Authors:** Eric A. Gbessi, Offianan A. Toure, Albert Gnondjui, Tossea S. Koui, Baba Coulibaly, Berenger A. Ako, Nguessan L. Tiacoh, Serge-Brice Assi, Ibrahima Sanogo, Didier-Paulin Sokouri, Ronan Jambou

**Affiliations:** 1 Unité de Paludologie, Institut Pasteur Côte d’Ivoire 01 BP 490 Abidjan 01 Côte d’Ivoire; 2 Institut Pierre Richet/Programme National de Lutte contre le Paludisme BP 1500 Bouaké Côte d’Ivoire; 3 Service d’Hématologie, CHU Youpougon Abidjan Côte d’Ivoire; 4 Département de santé globale, Institut Pasteur Paris 25 rue du Dr Roux 75015 Paris France; 5 Université Félix-Houphouet Boigny, Unité de Formation et de Recherche, Biosciences Abidjan Côte d’Ivoire

**Keywords:** Hemoglobinopathy, malaria, Ivory Coast, artemisinin containing therapy

## Abstract

*Background*: Artemisinin-based treatment in malaria patients with abnormal hemoglobin may be ineffective because of their genetic particularity, which could lead to resistance. The main purpose of this study was to assess the effect of artemisinin derivatives on *in vivo* parasite clearance according to erythrocyte variants. *In vivo* response was investigated through retrospective data obtained over a 42-day artemether-lumefantrine/artesunate amodiaquine efficacy protocol conducted from 2012 to 2016. *Results*: A total of 770 patients in Côte d’Ivoire attending the hospitals of Anonkoua-koute (Abidjan), Petit Paris (Korhogo), Libreville (Man), Dar es salam (Bouaké), Ayamé and Yamoussoukro with acute uncomplicated falciparum malaria were selected for successful hemoglobin typing. HbAS, HbSS, HbAC, and HbSC genotypes were found. Parasite clearance time was obtained for 414 patients. In the population with abnormal hemoglobin, parasite densities on admission and parasite clearance rates were significantly lower in the HbSC group compared to HbAA (*p* = 0.02 and *p* = 0.007, respectively). After PCR correction on day 42, the acute treatment rate was 100% for each group. Parasite half-life and time for initial parasitaemia to decline by 50 and 99% were longer for the HbSC group (*p* < 0.05). The study also investigated the prevalence of *K13-propeller* polymorphisms across different hemoglobin genotype groups. A total of 185 and 63 samples were sequenced in the HbAA group and patients with abnormal Hb, respectively. Only two nonsynonymous mutations *D559N* and *V510M* were found in the HbAA group. *Conclusion*: Although this study proved good efficacy of artemether-lumefantrine and artesunate amodiaquine in the treatment of uncomplicated *Plasmodium falciparum* malaria in patients with abnormal hemoglobin, the increased delay of parasite clearance may represent a threat to health in these patients in relation with sickle cell crisis, which could support selection of parasites resistant to artemisinin.

## Abstract (fr)

*Contexte* : Le traitement à base d’artémisinine chez les patients atteints de paludisme et présentant une hémoglobine anormale peut être inefficace en raison de leur particularité génétique, ce qui pourrait entraîner une résistance. L’objectif principal de cette étude était d’évaluer *in vivo* l’effet des dérivés de l’artémisinine sur la clairance du parasite en fonction des variantes érythrocytaires. La réponse *in vivo* a été étudiée à travers des données rétrospectives obtenues au cours d’un protocole d’efficacité de 42 jours artéméther-luméfantrine/artésunate-amodiaquine mené dans les années 2012 à 2016. *Résultats* : Un total de 770 patients en Côte d’Ivoire fréquentant les hôpitaux d’Anonkoua-koute (Abidjan), Petit Paris (Korhogo), Libreville (Man), Dar es salam (Bouaké), Ayamé et Yamoussoukro, présentant un paludisme aigu non compliqué à falciparum ont été sélectionnés pour un typage réussi de l’hémoglobine. Les génotypes HbAS, HbSS, HbAC et HbSC ont été trouvés. Le temps de clairance du parasite a été obtenu pour 414 patients. Dans la population avec une hémoglobine anormale, les densités parasitaires à l’admission et le taux de clairance parasitaire étaient significativement plus faibles dans le groupe HbSC par rapport au groupe HbAA (respectivement, *p* = 0,02 et *p* = 0,007). Le RCPA était de 100 % pour chaque groupe après correction par PCR au jour 42. La demi-vie du parasite et le temps nécessaire pour que la parasitémie initiale diminue de 50 et 99 % étaient plus longs pour le groupe HbSC (*p* < 0,05). L’étude a également examiné la prévalence des polymorphismes du gène K13 dans différents groupes de génotype d’hémoglobine. Un total de 185 et 63 échantillons ont été séquencés respectivement dans le groupe HbAA et chez les patients présentant une Hb anormale. Seules deux mutations non synonymes *D559N* et *V510M* ont été trouvées dans le groupe HbAA. *Conclusion* : Bien que cette étude ait prouvé la bonne efficacité de l’artéméther-luméfantrine et de l’artésunate amodiaquine dans le traitement du paludisme simple à *Plasmodium falciparum* chez les patients présentant une hémoglobine anormale, le retard accru de clairance parasitaire peut représenter une menace pour la santé de ces patients en relation avec la crise drépanocytaire, et peut favoriser la sélection de parasites résistants à l’artémisinine.

## Introduction

Hemoglobinopathies are inherited globin disorders [24] that affect 7% of the world population. 300,000 to 400,000 children are born each year with a severe form of the disease [28]. When glutamin is replaced by valin (*6GAG > 6GTG*), hemoglobin is called HbS (Hemoglobin S) and when replaced by lysine (*6GAG > 6AAG*), the abnormal hemoglobin is *HbC* (Hemoglobin C) [24]. In West Africa, the main mutation results in S or C variants of hemoglobin.

In addition to studies on thalassemia that have shown protection against malaria [11, 31] other studies focusing on HbS and HbC have shown that these abnormalities are involved in protection against malaria [2, 3, 13, 14]. This protection concerns not only malaria infection itself but mainly severe forms of malaria. Clinical responses of patients with hemoglobinopathy can differ according to host genetics [3, 7]. Although there is no difference in chloroquine metabolism, results published by Orjih in 1987 indicated that the hemoglobin genotype modulates the level of chloroquine in erythrocyte cells [16]. Another study showed that artesunate and chloroquine had less activity against *Plasmodium falciparum* growing in alpha-thalassemia and/or HbCS (Hb Constant Spring) erythrocytes than in normal erythrocytes, while the activity of pyrimethamine was the same for all erythrocytes. This could be due to an increased amount of antioxidant enzymes naturally found in abnormal erythrocytes, which could counterbalance the oxidative activity of artesunate [9, 32]. This suggests that patients with hemoglobinopathies may be at increased risk of inadequate response to artemisinin combination therapies (ACTs), especially in areas where parasite resistance or tolerance to artemisinin derivatives have emerged [18]. These results highlight the important role of hemoglobinopathy in parasite-host-drug interactions. However, very few studies address this question of the effects of erythrocyte variants on antimalarial efficacy. The 50% inhibitory concentration (IC50) of artesunate in normal and abnormal cells is used to assess its efficacy *in vitro*, and this concentration was found to be higher in abnormal cells [32]. *In vivo,* this efficacy can be measured by parasite clearance expressed as the parasite half-life in the patients’ blood. Using this parameter, the usual time to denote artemisinin resistance is 5 h [8].

A recent study in Ghana found that the parasite clearance in sickle cell disease (SCD) children with uncomplicated malaria was slower compared with clearance in non-SCD children, although artesunate-amodiaquine (AA) and artemether-lumefantrine (AL) showed similar clinical and parasitological effects in the SCD and non-SCD groups [1]. The World Health Organization has endorsed ACT as first‐line treatment where the potentially life‐threatening parasite *Plasmodium falciparum* is the predominant infecting species [6]. This strategy has been implemented in Côte d’Ivoire since 2005 [22], where the proportion of patients with abnormal hemoglobin attending dispensaries for malaria is largely unknown and probably underestimated. *HbS* and *HbC* genotypes were found in all the regions of Côte d’Ivoire with the highest prevalence (27%) in the Northern region [20].

The main purpose of this study was to assess *in vivo* the effect of artemisinin on parasite clearance according to erythrocyte variants. Data collected during *in vivo* drug efficacy surveys conducted on behalf of the National Malaria Control Program were analyzed retrospectively.

## Materials and methods

### Ethical considerations

Studies were conducted at all sites according to the declaration of Helsinki and national legal and regulatory requirements. Protocol, case report form, and informed consent form were approved by the “Comité National d’Éthique de la Recherche” of Côte d’Ivoire [21].

### Study sites

**Figure 1:**
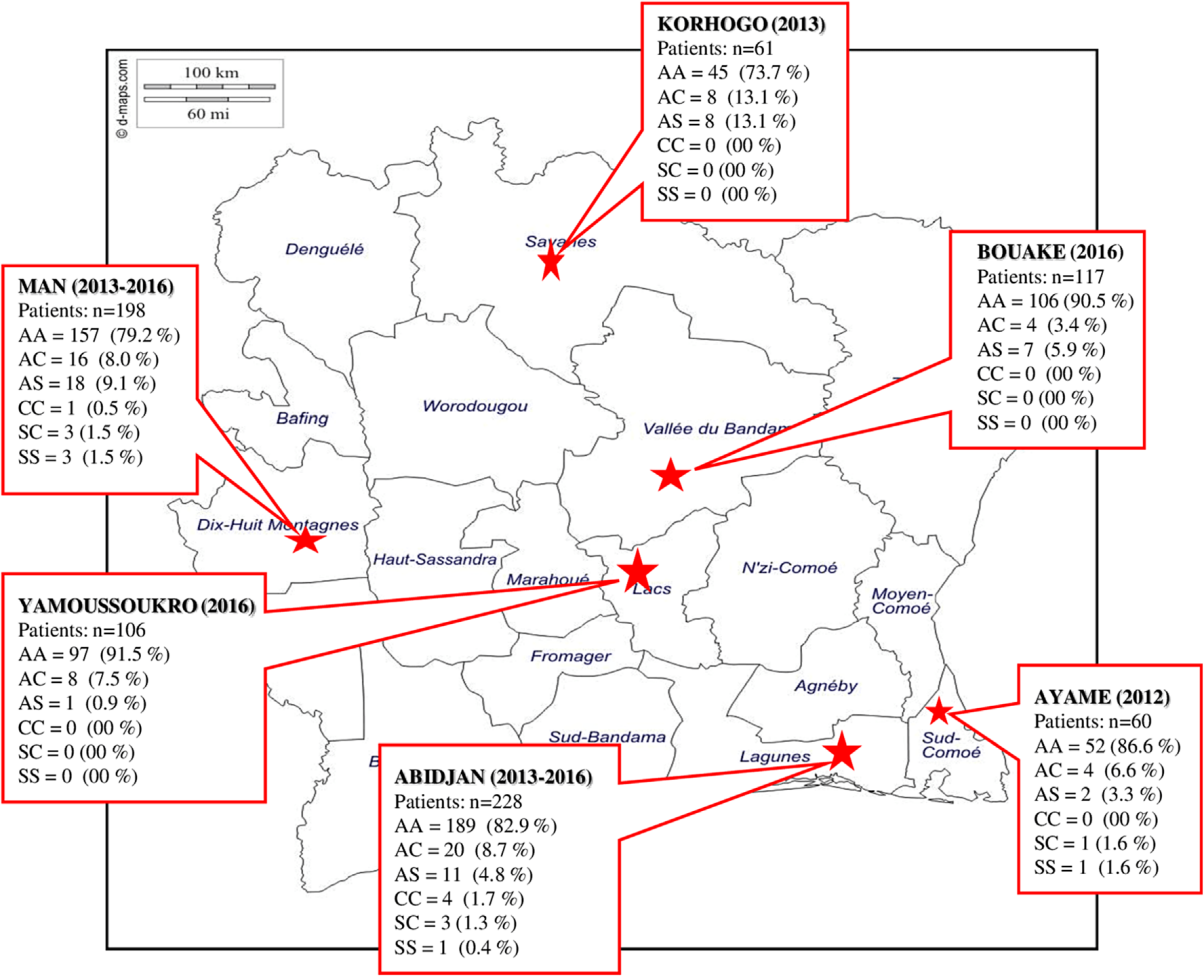
Map of Côte d’Ivoire with site locations and prevalence of hemoglobin phenotypes.

During the past 8 years, six study sites were investigated in the country (Fig. 1). They are located in various ecological contexts, which can induce different levels of premunition and thus of parasitemia. Abidjan is the economic capital of the country accounting for almost 6 million inhabitants. It is located in the South of the country, with a sub-equatorial climate. Ayamé is located 100 km North–East of Abidjan, in a region of lakes. It is a tropical area where rainfalls almost all year and with a very high malaria transmission level. Yamoussoukro is the administrative capital, 248 km North of Abidjan, located in a region of lakes with an equatorial climate. Transmission of malaria is lower with rainfall of less than 900 mm/year. Man is the main town of the Western part of the country, 150 km from Yamoussoukro, surrounded by hills and dense forests. The climate is tropical and transmission of malaria is intense. Bouaké has 1 million inhabitants and is located 200 km North of Yamoussoukro, located in a savannah region. Korhogo is on the border with Mali and Burkina Faso. In these two latter places, the climate is typically sub-Sahelian (dry and hot) and malaria transmission is less intense than in other parts of the country [20].

### Study population

The patients included in this study were recruited during therapeutic efficacy studies of ACTs, conducted on behalf of the National Malaria Control Program. Protocol of enrolment followed the 42 days WHO protocol [29].

Patients with microscopically confirmed acute uncomplicated falciparum malaria, parasite density ranging from 2000 to 200,000 asexual parasites/μL (both inclusive) and an axillary temperature ≥ 37.5 °C or history of fever in the past 24 h, were included in the study. Exclusion criteria included general signs of severe falciparum malaria according to the WHO definition. Patients with other plasmodium species than falciparum were not included, as well as patients already treated before attending the dispensary or patients unable to take oral medication.

Patients attending dispensaries for malaria suspicion were clinically examined and malaria was confirmed by Giemsa-stained thick smear examination. Patients received either AA or AL for 3 days under supervision by medical staff. Follow-up of patients through clinical investigation and blood sample was done during systematic outpatient visits at days 1, 2, 3, 7, 14, 21, 28, 35, and 42. Patients recruited in Ayamé and Bouaké were hospitalized for 3 days and followed-up at days 3, 7, 14, 21, 28, 35, and 42. During the first 3 days, thick blood smears were done every 6 h until two consecutive negative parasitemia results were obtained [22, 29]. Treatment failure was confirmed by microscopy examination of thick blood smears and defined either as “parasitological failure” if parasites are detected on the thick smear, or as “clinical failure” in case of presence of parasites on the thick smear plus fever. Early clinical failure, late clinical failure, and parasitological failure were defined as previously [29]. For all the patients with positive smear, blood samples were taken using EDTA-tubes and 50 μL were blotted on to Whatman 3 MM cards. Isolates from patients with treatment failure (but not those with treatment success) were genotyped using msp1 and msp2 nested-PCR to exclude new infection [29].

### Hemoglobin typing

Two methods of hemoglobin typing were used according to the samples available. For samples from Yamoussoukro, Ayamé, and Korhogo, electrophoresis of hemoglobin was performed on Hydrasys^®^ gel, following the manufacturer’s recommendations [23]. For blood samples collected on paper, typing was done using the FRET (fluorescence resonance energy transfer) approach after DNA extraction and PCR amplification [20].

### *K13-propeller* genotyping and sequencing

DNA was extracted using a QiAamp^®^ DNA kit, according to the manufacturer’s recommendations. DNA was stored at −20 °C. *Plasmodium falciparum* K13 gene was amplified and bi-directionally sequenced using the Sanger protocol by Genewiz institute. Sequences were aligned with the K13 *P. falciparum* 3D7 reference sequence (PF3D7-1343700) [4].

### Statistical analysis

The number of patients enrolled by site was set as recommended by the WHO protocol [29]. At least 50 patients were registered for each arm (AA or AL). For 6 h and 24 h follow-up, parasite clearance was analyzed with the *Antimalarial Resistance Network parasite clearance estimator* (https://www.wwarn.org) and the *WHO online parasite clearance estimator* (http://www.who.int/malaria/areas/treatment/drug_efficacy/en/), respectively. For each group of patients with normal or abnormal hemoglobin, the time required for the decrease of X% of the initial parasitemia (PCx) was calculated. The *parasite half-life* was defined as the time required for the number of parasites in the peripheral blood (parasitemia) to decrease by 50%. It is derived from the slope of the linear part of the log-transformed parasitemia versus time curve. The clearance rate (*k*) was defined as the decrease of parasitemia per unit of time (per hour). Decreased response of treatment or the potential resistance of parasites are defined as the presence of parasites in blood on day 3 or by parasite half-life greater than 5 h. Pearson’s *χ* ^2^ test and Fisher’s exact test were used for comparison of proportions, whereas the Mann–Whitney test was used for parasite densities and parasite clearance data. Descendant logistic regression was used to define risk factors associated with the occurrence of treatment failures with *p*-value > 0.2 as cut-off of exclusion of the variables. Parameters used included region, parasitemia at day 0 (D0), gender, age, type of hemoglobin and parasitemia decay (quoted as more or less 5 h). Data analysis was done on R, version 3 environment and GraphPad Prism version 5. A *p*-value ≤ 0.05 was considered statistically significant.

## Results

### Population enrolled and parasitemia

Samples from 770 patients with uncomplicated malaria were analyzed for hemoglobin genotyping. Among them, 124 patients (16.1%) harbored abnormal hemoglobin (aHb). The aHb genotype distributions of these patients were: *AC*, *n* = 60 (7.79%); *AS*, *n* = 47 (6.10%); *CC*, *n* = 5 (0.64%); *SC*, *n* = 7 (0.90%) and, *SS*, *n* = 5 (0.64%). Patient mean age and the number of patients under 5 years old were similar in all groups (*p* > 0.05) (Table 1). Unlike patients with *AC*, *AS* and *CC* genotypes, parasite density at enrolment was lower in patients with *SC* genotype in comparison to the AA group (*p* = 0.02).

**Table 1:** Baseline demographic characteristics.

	HbAA	HbAC	HbAS	HbCC	HbSC	HbSS
Characteristics at day 0 *n* (%)	646	60	47	5	7	5
Age (years), mean (±*SD*)	12.7 (12.9)	13.5 (9.7)	14.9 (15.9)	11.0 (5.3)	12.7 (7.6)	10.8 (8.6)
Age (<5 years), *n* (%)	177 (27.4)	13 (21.6)	14 (29.7)	0 (00)	0 (00)	2 (40.0)
Sex, *n* (%) females	344 (53.2)	30 (50)	27 (57.4)	4 (80.0)	4 (57.1)	2 (40.0)
Pf parasitemia (/μL of blood), median (IQR)	28,978 (7982–72,000)	20,052 (5980–68,156)	17,280 (4644–82,400)	27,394 (25,000–34,690)	8400 (3654–16,650)	15,700 (14,892–24,000)
*p*-values		HbAA *vs.* HbAC	HbAA *vs.* HbAS	HbAA *vs.* HbCC	HbAA *vs.* HbSC	HbAA *vs.* HbSS

Age (years), mean (±*SD*)		0.07	0.58	0.55	0.41	0.93
Age (<5 years), *n* (%)		0.36	0.73	0.33	0.19	0.61
Sex, *n* (%) females		0.68	0.65	0.37	1	0.69
Pf parasitemia (/μL of blood), median (IQR)		0.15	0.40	0.97	0.02	0.56

*Abbreviations*: Hb, hemoglobin; Pf., *Plasmodium falciparum*; *SD*, standard deviation; μL, microliter; IQR, interquartile range.

### Parasite clearance

Parasite clearance was estimated for 414 patients. In this study, the median parasite clearance time (PCT) varied between 24 and 48 h, with 24 h for *AA, AC, AS* and *SS* genotypes against 48 h for *CC* and *SC* genotypes. In general, there was no difference between the PCT of normal hemoglobin (nHb) compared to abnormal hemoglobin (aHb) (nHb: 32.6 h ± 12.5; aHb: 32.3 h ± 12.8, *p* = 0.68). Specifically, the PCT was thus longer for patients with *CC* and *SC* genotypes than for *AA* genotype, where differences were only significant between *SC* and *AA* groups (*p* = 0.02). Patients with HbSC, had a longer parasite half-life time (clearance 50%) (*p* = 0.008) and a lower parasite clearance rate (*p* = 0.007) than those with AA hemoglobin (Table 2). Proportion of isolates potentially resistant, proportion of patients with parasitemia at days 3 > 10% of the day 0 (Table 3), and the number of patients with a slope half-life > 5 h (Table 2), were not different for patients with or without aHb (*p* = 1). Acute clinical and parasitological response (ACPR) rates on day 42 were similar in all groups (100%) (Table 3).

**Table 2:** Characteristics of parasite clearance according to hemoglobin type.

	HbAA	HbAC	HbAS	HbCC	HbSC	HbSS
Patients for parasite clearance calculation (*n*)*	359	20	22	4	6	3
Median *k* (/hour), (IQR)	0.2 (0.2–0.3)	0.2 (0.2–0.2)	0.2 (0.2–0.3)	0.2 (0.1–0.2)	0.1 (0.1–0.2)	0.2 (0.1–0.2)
Slope half-life (hour), median (IQR)	3.1 (2.7–3.6)	2.9 (2.7–3.2)	2.6 (2.3–3.6)	3.4 (3.2–3.6)	3.8 (3.6–3.9)	3.2 (2.5–4.3)
Patients with slope half-life > 5 h, *n* (%)	26 (7.2)	1 (5.0)	1 (4.5)	0	0	0
PCT in hours, median (IQR)	24 (24–48)	24 (24–36)	24 (24–48)	48 (42–48)	48 (48–48)	24 (24–48)
PC50, median (IQR)	12.1 (9.4–13.3)	11.9 (8.2–12.4)	11.7 (9.2–13.9)	12.8 (12.5–13.1)	13.7 (13.6–14.0)	12.5 (9.2–14.4)
PC90, median (IQR)	21.9 (14.7–23.7)	21.7 (14.5–22.6)	20.3 (13.3–23.9)	23.4 (22.9–23.9)	24.8 (24.2–25.1)	22.9 (15.0–26.2)
PC95, median (IQR)	24.9 (17.3–27.0)	24.8 (17.3–25.7)	23.2 (15.1–27.3)	26.7 (26.1–27.3)	28.3 (27.6–28.6)	26.1 (17.5–29.9)
PC99, median (IQR)	31.0 (23.0–33.6)	30.7 (23.7–31.9)	28.8 (19.2–33.9)	33.1 (32.3–33.8)	35.0 (34.2–35.4)	32.3 (23.4–36.9)
*p*-values		HbAA *vs.* HbAC	HbAA *vs*. HbAS	HbAA *vs.* HbCC	HbAA *vs.* HbSC	HbAA *vs.* HbSS

Median *k* (/hour), (IQR)		0.44	0.80	0.16	0.007	0.54
Slope half-life (hour), median (IQR)		0.89	0.87	0.18	0.008	0.56
PCT in hours, median (IQR)		0.13	0.49	0.05	0.02	0.82
PC50, median (IQR)		0.34	0.74	0.24	0.006	0.65
PC90, median (IQR)		0.56	0.83	0.13	0.01	0.53
PC95, median (IQR)		0.55	0.82	0.12	0.02	0.14
PC99, median (IQR)		0.64	0.86	0.15	0.03	0.54

*Notes*: PC50, 50% parasite clearance rate; PC90, 90% parasite clearance rate; PC95, 95% parasite clearance rate; PC99, 99% parasite clearance rate.

* Parasite clearance was estimated by WWARN PCE and WHO PCE.

*Abbreviations*: Hb, hemoglobin; IQR, interquartile range; PCE, parasite clearance estimator; PCT, parasite clearance time; *k*, parasite clearance rate.

**Table 3:** Characteristics of patients during follow-up according to hemoglobin type.

	HbAA	HbAC	HbAS	HbCC	HbSC	HbSS
Patients followed-up from day 3 onward (*n*)	646	60	47	5	7	5
Positive parasitemia on day 3 *n* (%)	3 (0.5)	1 (1.7)	0 (00)	0 (00)	0 (00)	0 (00)
Early treatment failure (ETF) *n* (%)	0 (00)	0 (00)	0 (00)	0 (00)	0 (00)	0 (00)
Late clinical failure (LCF) *n* (%)	33 (5.1)	4 (6.7)	1 (2.1)	1 (20)	0 (00)	0 (00)
Late parasitological failure (LPF) *n* (%)	7 (1.1)	0 (00)	0 (00)	0 (00)	0 (00)	0 (00)
Recrudescence (defined on PCR typing) *n* (%)	0 (00)	0 (00)	0 (00)	0 (00)	0 (00)	0 (00)
New infection *n* (%)	40 (6.2)	4 (6.7)	1 (2.1)	1 (20)	0 (00)	0 (00)
Adequate clinic parasitological response (ACPR) (%)	100	100	100	100	100	100
*p*-values		HbAA *vs.* HbAC	HbAA *vs.* HbAS	HbAA *vs.* HbCC	HbAA *vs.* HbSC	HbAA *vs.* HbSS

Positive parasitemia on day 3 *n* (%)		0.29	1	1	1	1
Early treatment failure (ETF) *n* (%)		1	1	1	1	1
Late clinical failure (LCF) *n* (%)		0.54	0.72	0.23	1	1
Late parasitological failure (LPF) *n* (%)		1	1	1	1	1
Recrudescence (defined on PCR typing) *n* (%)		1	1	1	1	1
New infection *n* (%)		0.78	0.35	0.27	1	1

**Figure 2:**
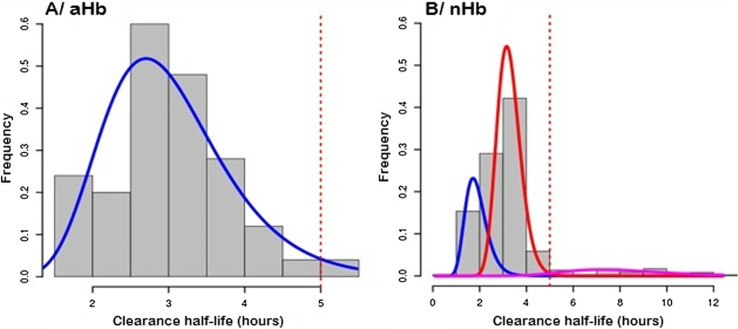
Modeling the population parasite number according to the hemoglobin type. The parasite half-life time is plotted for those with (A) or without (B) abnormal hemoglobin according to the White et al. [27]*,* distribution model. For aHb, the model describes only one phenotype (half-life time = 2.15 ± 1.37 h), whereas for nHb, three phenotypes were found: i) phenotype 1 (blue line, half-life time = 1.83 ± 1.28 h) accounting for 25.4% of the patients, ii) phenotype 2 (red line, half-life time = 3.23 ± 1.17 h) accounting for 66.88% of the patients, and iii) phenotype 3 (pink line, half-life time = 7.78 ± 1.32 h) accounting for 7.73%. The red line highlights the threshold of potential resistance (half-life more than > 5 h). aHb (Abnormal hemoglobin), nHb (Normal hemoglobin).

However, when time for parasite half-life time of each patient is plotted as histogram and analyzed according to White et al. [27], different distributions of parasite population can be highlighted (Fig. 2): i) for patients with aHb, only one parasite population with a mean half-life time at 2.15 ± 1.37 h (sensitive to ACT), ii) for patients with nHb three populations of parasites can be predicted with half-life time at 1.83 ± 1.28 h for 25.4% of the patients, 3.23 ± 1.17 h for 66.9% of the patients, and 7.78 ± 1.32 h for 7.7% of the patients (Fig. 1). This last group can be considered “ACT low sensitivity”.

### Parameters linked to drug failure

A logistic regression model was used to search for parameters linked with the risk of treatment failure (both parasitological LPF and clinical failure ETF/LCF). The model stopped with Wald test at 31.84 (4 *df*, *p* = 2.066e−06). Only the age group (age < 5 years, odds ratio [OR = 5.74], *p* < 0.0001), the half-life of parasite decay more than 5 h (OR = 3.69, *p* = 0.01) and HbCC genotype (OR = 11.74, *p* = 0.04) were significantly linked with the risk of failure (Table 4).

**Table 4:** Multivariate analysis of risk factors associated with the occurrence of treatment failures.

Logistic model	Failure (%)	Odds ratio [CI 95%]	*p*-value
Half-life (hours)			
< 5 h	40 (10.4)	1	–
> 5 h	6 (21.4)	3.69 [1.18–10.3]	**0.01**
Hemoglobin type			
AA	40 (6.2)	1	–
AC	4 (6.7)	1.5 [0.21–6.25]	0.62
AS	1 (2.1)	0.54 [0.02–2.94]	0.56
CC	1 (20.0)	11.74 [0.54–104]	**0.04**
SC	0 (0.0)	0	0.99
SS	0 (0.0)	0	0.99
Gender			
Female	18 (4.4)	1	–
Male	28 (7.8)	1.57 [0.72–3.53]	0.25
Age group			
≥ 5 years	16 (2.8)	1	–
< 5 years	30 (14.6)	5.74 [2.6–13.1]	**0.0001**

*Notes*: Logistic regression stopped with Wald test 31.84 on 4 *df* (*p* = 2.066e−06). Only the variables age group (age < 5 years), parasite half-life, and HbCC were significantly linked with the risk of failure.

### *K13-propeller* genotypes

Overall, 247 sequences were obtained for the *K13-propeller* gene, 63 sequences for aHb and185 sequences for nHb. These sequences derived from parasite isolates with density greater than 10,000/μL of blood. Height mutations were detected out of which only two were non-synonymous (*D559N* and *V510M*). These non-synonymous mutations were found in isolates from people with nHb. Only one synonymous mutation was found in isolates from patients with aHb (1.61%) (Table 5).

**Table 5:** Prevalence of K13*-propeller* gene mutations according to hemoglobin type.

	HbAA	HbAC	HbAS	HbCC	HbSC	HbSS
Sequences obtained (*n*)	185	27	22	05	04	05
Sequences WT, *n* (%)	178 (96.2)	24 (100)	22 (100)	05 (100)	04 (100)	04 (80.0)
Mutated sequences, *n* (%)	07 (3.8)	0 (00)	0 (00)	0 (00)	0 (00)	01 (20)
Nature of mutation						
Synonymous mutations, *n* (%)	05 (2.70)	–	–	–	–	01 (20)
Non-synonymous mutations, *n* (%)	02 (1.08)	–	–	–	–	–

*Notes*: Synonymous mutations (*HbAA*: Y519Y R597R V510V T535T C469C, *HbSS*: C469C); Non-synonymous mutations (*HbAA*: D559N V510M).

*Abbreviations*: WT, wild type; Hb, hemoglobin.

## Discussion

This study focuses on parasite clearance in patients with hemoglobin disorders. The *in vivo* effect of ACTs on parasites was evaluated in patients who attended consultation without prior knowledge of their hemoglobin status. From 2012 to 2016, two protocols of follow-up were used on behalf of the National Malaria Program to survey drug efficacy, for a 24-hour follow-up and a 6-hour follow-up.

For the 770 patients enrolled in this study in 6 provinces, a significantly lower initial parasite density was found in *HbSC* genotype patients, compared with *HbAA* group, as previously reported [7, 19, 26]. According to the literature, this was related to a lack of maturation of parasites in sickle cells, an increase in phagocytosis of infected red blood cells, or more recently, to the effect of human microRNA on the intracellular control of the parasite [14, 20]. In this study, during treatment, a lower clearance rate, a longer time to 50% and 99% parasite clearance, and a longer parasite half-life were found for people with *HbSC* than for *HbAA* patients, but not for other types of hemoglobin. Previous *in vitro* results have shown that artesunate and chloroquine had less activity against *P. falciparum* growing in alpha-thalassemia and/or *HbCS* erythrocytes than normal erythrocytes, while the activity of pyrimethamine was the same for all erythrocytes [32]. Clearly, these data support evidence that hemoglobinopathies modulate the activity of artemisinin derivatives. Authors attribute these results to altered accumulation and lower binding of molecules within the parasitized erythrocytes as well as attenuation of oxidative stress [17, 18]. Antagonistic actions of erythrocyte antioxidative enzymes [17] and artesunate oxidative activity [5] could lead to this higher clearance time in abnormal erythrocytes. However, the low initial parasite density found in these patients with *HbSC* genotype could have led us to expect even faster parasite clearance. These results show that normal and abnormal blood cells have significantly different biochemistry.

However, the best measure of parasite clearance is the parasite half-life in a patient’s blood. The usual threshold used for artemisinin resistance is 5 h. This definition complements the WHO definition arguing that artemisinin resistance must be suspected in a population if more than 10% of patients continue to carry parasites 3 days after beginning of ACT treatment [27]. Globally, this study showed no failure difference between patients with or without aHb. The same applies for the various therapeutic failure criteria (ET, LC, LPF). Indeed, in logistic regression, the main factors associated with failure were, parasite half-life time “> 5 h”, group of “age < 5 years”, and homozygote “*HbCC* genotype”.

This last parameter is interesting as *HbC* is mainly a Sahelian genetic trait and two studies demonstrated the malaria protective effect of this hemoglobin. Agarwal et al. studying a Dogon population with a high prevalence of *HbC*, reported that subjects with *HbAC* were 29% less likely to develop severe malaria than subjects with *HbAA* [2]. One year later, in Burkina Faso, a study confirmed that *HbC* was associated with about 90% reduced risks of malaria (both mild and severe) [14]. Recently, a study implemented in Mali, indicated that schoolchildren with hemoglobin C mutation might contribute disproportionately to the seasonal malaria resurgence in an area where the *HbC* variant is common, related to an increase of asymptomatic carriage of parasites [10]. Data obtained during this study in Côte d’Ivoire support this last point and add rational to additional effects of *HbC* on malaria epidemiology, with delayed efficacy of artemisin derivatives (ART) for these patients.

Artemisinin resistance monitoring remains a priority for the National Malaria Control Program of Cote d’Ivoire because of the threat of import of resistant strains from Asian countries [15, 30]. Genetic analysis of populations of parasites is thus an important tool to conduct retrospective surveys. The *A578S* mutation observed in Yunnan province, China associated with the predominant *F446I* mutation [19], has already been reported in some African countries [12]. In this study, two new nonsynonymous mutations (*D559N* and *V510M*) were found in patients with nHb, not related to Southeast Asia ones [19], without association with abnormal hemoglobin or delay response. However, at the same time, an analysis conducted by White et al. [25, 27] supports different structures of parasite populations for patients with aHb and nHb. This could suggest higher biochemical constraints for the development of the parasite in aHb. Interestingly, in abnormal cells, constraints in parasite growth significantly reduce biomass and therefore can affect the phenotypic diversity of parasites. The second hypothesis could be that aHb can selects specific phenotypes usually at low frequency in the parasite population. Given the high frequency of people living with aHb in Côte d’Ivoire, this could place considerable pressure on parasites, supporting emergence of drug resistance (as resistance to ART seems related to anti-oxidant metabolism).

In conclusion, despite the significant delay in parasitic clearance observed in HbSC patients compared to HbAA patients, the ACTs remain highly effective in these patients. This is good news for the National Malaria Control Program, as patients with any type of hemoglobin will be able to use the same antimalarial drug. However, the high homogeneity of the isolates invading aHb patients suggests a specific genetic background in these parasites. Full genome sequencing of these isolates is now in progress.
